# Software-defined microwave photonic filter with high reconfigurable resolution

**DOI:** 10.1038/srep35621

**Published:** 2016-10-19

**Authors:** Wei Wei, Lilin Yi, Yves Jaouën, Weisheng Hu

**Affiliations:** 1 State Key Lab of Advanced Optical Communication Systems and Networks, Shanghai Jiao Tong University, 200240 Shanghai, China; 2 LTCI CNRS, Télécom ParisTech, Université Paris Saclay, 75013 Paris, France

## Abstract

Microwave photonic filters (MPFs) are of great interest in radio frequency systems since they provide prominent flexibility on microwave signal processing. Although filter reconfigurability and tunability have been demonstrated repeatedly, it is still difficult to control the filter shape with very high precision. Thus the MPF application is basically limited to signal selection. Here we present a polarization-insensitive single-passband arbitrary-shaped MPF with ~GHz bandwidth based on stimulated Brillouin scattering (SBS) in optical fibre. For the first time the filter shape, bandwidth and central frequency can all be precisely defined by software with ~MHz resolution. The unprecedented multi-dimensional filter flexibility offers new possibilities to process microwave signals directly in optical domain with high precision thus enhancing the MPF functionality. Nanosecond pulse shaping by implementing precisely defined filters is demonstrated to prove the filter superiority and practicability.

In radio frequency (RF) systems, the microwave photonics (MWP) has been attracting persistent attentions since it provides potential possibilities to overcome some inherent limitations of electronics^1^,^2^,^3^. As a key MWP component, the microwave photonic filter (MPF) has been studied intensively and has shown its prominent superiority in terms of tunability and reconfigurability^4^,^5^. By using MPFs instead of conventional electrical filters, the RF system flexibility can be increased dramatically. The realization of the MPF can be roughly categorized into two approaches. One is delayed-tap based finite impulse response (FIR) filter. For this method, the RF signal is first modulated on several optical carriers at different wavelengths. Each carrier acts as a filter tap and the tap coefficients are usually set by amplitude and phase manipulation with liquid crystal based technique^6^,^7^. Then the RF-modulated carriers pass through dispersive media to obtain different time delay and are combined together to convert to RF signal. The optical carriers can be multiple laser diodes^8^, a broadband optical source^6^, an optical comb^7^,^9^ or a multi-mode mode lock laser^10^. The dispersive media can be a dispersive fibre^11^ or a chirped fibre Bragg grating (CFBG)^12^. This method provides certain flexibility but due to the inherent nature of the discrete FIR filter, the frequency response has multiple harmonic passbands, which restricts the filtering within one free spectral range (FSR). Several works have been reported to retain a single passband including employing an optical filter^7^ and broadening the tap width^13^. The other category is using optical filter techniques to filter the RF-modulated optical signal directly by micro-ring resonators^14^, fibre Bragg grating^15^, liquid crystal arrays^16^ and stimulated Brillouin scattering (SBS)^17^. Meanwhile, in order to enhance functionality and robustness and reduce cost, size and power consumption, there has been a consistent effort towards the integration of microwave photonic devices^18^,^19^,^20^,^21^.

SBS effect can be considered as an active optical filter in terms of signal selection. Several SBS-based MPFs have been reported since a decade ago. Thanks to the narrow SBS linewidth, the SBS-based MPFs have a very high discrimination resolution^17^. The filter tunability is easily realized by modulating a single tone signal at different frequency^22^,^23^ or by using a second laser source^24^. The filter selectivity can be further increased by using phase modulated probe^25^ or double sideband cancellation^26^,^27^,^28^. The filter bandwidth is reconfigurable by broadening the Brillouin pump with different modulation schemes^29^,^30^. Moreover, the filter shape can be tailored if the modulated electrical waveform is well designed^31^,^32^,^33^,^34^,^35^. SBS can also be used for realizing tap-based MPFs by manipulating the phase of the signal^36^ or employing dynamic Brillouin gratings^37^. Furthermore, the progress of the integrated Brillouin improves the filter compactness^38^,^39^. All these distinct advantages make it a promising candidate for MPFs. We list several representative works for SBS based MPFs in Table 1 with critical parameters for comparison.

The existing programmable or reconfigurable MPF solutions can provide bandwidth and central wavelength tunability. But as for the filter shape, most of the MPFs are not capable of reconfiguring it or only provide reconfigurability with resolution of several hundred MHz due to the limitation of tap control or optical filtering resolution^16^,^40^,^41^,^42^. Although the SBS-based MPFs have potential for shape tailoring, the pump control methods mentioned above have their inherent resolution limitations. Thus no arbitrary-shaped filter with ~MHz resolution has been demonstrated yet. Therefore generally the MPF is used more for signal selection up to now other than real signal processing. For easy comparison, we also list several representative shape-reconfigurable MPFs in Table 1 with some critical parameters.

Previously, we have proposed a rectangular optical filter approach based on SBS effect in fibre^43^. We realized a precise and accurate Brillouin pump control scheme adopting electrical broadband signal generation and digital feedback correction. We demonstrated rectangular optical filters with tunable bandwidth from 50 MHz to 4 GHz and passband ripple of ~1 dB. By employing dual-stage pump configurations, the filter selectivity reached ~40 dB and the noise performance was also improved^44^. The filter feasibility has been proved by its application in a small-grid reconfigurable optical add and drop multiplexer (ROADM) where the proposed filters were used to precisely amplify and absorb a single sub-band from a super-channel signal^45^,^46^. Furthermore, we demonstrated a polarization-independent rectangular MPF approach with selectivity as high as 57 dB by using a depolarized sweeping pump^47^. However, in the previous work, only rectangular filter shape has been realized, which is still far from the requirement of the arbitrary-shaped MPF.

In this paper, we realize software-defined MPF with arbitrary-shaped configurability thus offering possibilities to process microwave signal directly in optical domain with a very high precision. We first demonstrate the high controllability of the filter bandwidth and central frequency which can be changed with resolution of ~MHz. The main parameters are also listed in Table 1. Then we precisely define the pump shape by software and obtain arbitrary-shaped MPFs such as super-Gaussian, triangle, inverse Gaussian or combinations of these shapes. We further adopt depolarized sweeping pump to realize polarization-independent arbitrary-shaped MPFs. Finally we demonstrate the pulse shaping capability using precisely defined filters to obtain different pulse shapes. With multi-dimensional flexibility, the proposed MPF can find its versatile and unique applications in microwave photonic fields. Furthermore, this optical filtering technique can also be applied to optical communications and high-resolution optical signal processing.

## Results

### Pump design and filter shape control

The proposed SBS filter includes two key techniques: first the arbitrary-shaped pump generation and second the filter shape control. Both procedures are software-defined and can be controlled with very high precision.

**Figure 1:**
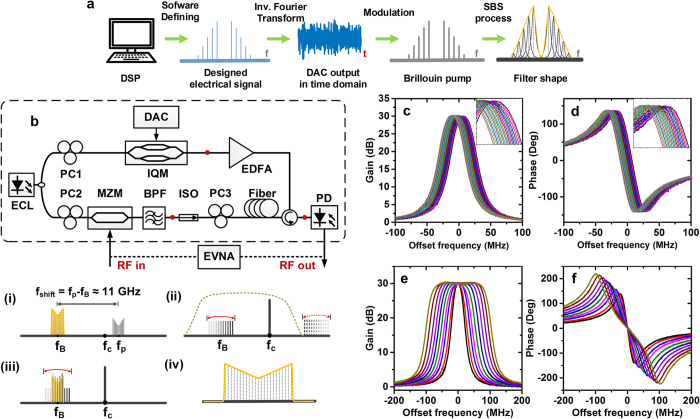
Filter generation process with inherent flexibility. (**a**) Principle of the pump spectral-shape design. (**b**) Experimental setup for measuring and adjusting filter shapes. Inset (i) single sideband pump fp, arbitrary-shaped SBS gain around fB and ECL laser frequency fc, (ii) sweeping probe signal, (iii) sweeping probe signal amplified by the SBS gain, (iv) measured gain spectrum. The amplitude (**c**) and phase (**d**) response of the MPF showing fine position tuning with a resolution of 1 MHz. The amplitude (**e**) and phase (**f**) response of the MPF showing fine bandwidth tuning with a resolution of ~20 MHz.

The natural SBS gain bandwidth ranges from 10 MHz~50 MHz depending on different types of fibres^48^. The effective Brillouin gain spectrum for a broadband pump can be obtained by convolving the natural Brillouin gain with the pump spectrum^49^. Intuitively, in order to obtain arbitrary-shaped SBS gain spectrum, a pump consisting of variable-amplitude spectral lines with interval equal to the natural SBS gain bandwidth is required. As shown in Fig. 1a, we first precisely define the targeted pump spectral-shape in frequency domain through a digital signal processor (DSP) by software. Then we obtain the corresponding time domain signal by using inverse Fourier transform and accurately generate the electrical waveform by a digital-analog converter (DAC). The designed electrical signal then modulates a CW light acting as the pump wave. Here we assume that the modulation is approximately linear by using a small-signal modulation thus ensuring the identity of the electrical driving signal and the optical pump. After boosted to a high power level, the pump gives rise to the SBS effect. If the signal is shifted downward from the pump to the Brillouin frequency, it will be amplified as the Stokes wave, and if it is shifted upward to the Brillouin frequency, it will be absorbed as the anti-Stokes wave^50^. This can be considered as filtering in terms of signal selection and transformation. It should be noted that the SBS gain is only related to the pump power which is proportional to the amplitude of electrical spectral lines, thus we just adjust the amplitude of each spectral line and set a random phase for each line to maintain an acceptable peak to average ratio of the waveform.

This pump generation approach differs from the previous proposed methods and brings about many distinctive benefits. First, the natural SBS bandwidth is only ~20 MHz, thus the minimum filter bandwidth can be very small and the controlling precision is very high. Second, the electrical signal is generated accurately by the DAC within a specific frequency range, ensuring the steep filter edges. Third, the amplitude of each comb line can be altered accurately by software to optimize the filter passband shape. Fourth, the number of the comb line can be changed to adjust the filter bandwidth precisely. Fifth, the filter central frequency can be shifted by tuning the wavelength of the comb electrically and optically. In conclusion, the proposed MPF based on this pump spectral-shape control method has the capability of reconfiguration in terms of bandwidth, central frequency and most importantly the shape with very high precision.

Given the nonlinear responses of electrical and optical components as well as the error from linear modulation estimation, there is always a deviation between the designed filter shape and the actual filter shape. In order to control the shape of the targeted SBS filter response accurately, a feedback compensation is required to digitally control the amplitude of each electrical spectral line according to the measured SBS gain shape. The feedback algorithm is based on the analytical relation between signal gain and pump power^49^:


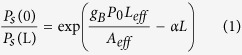


where Ps(0) and Ps(L) are the output and input power of the SBS filter (i.e. Ps(0)/Ps(L) is the signal gain), P_0_ is the pump power and αL is the fibre loss. We reasonably assume that the SBS gain at a certain frequency is only related to the corresponding electrical spectral line. Thus once the SBS gain shape has been obtained, we can use the derived relation between the SBS gain and the corresponding electrical frequency component to calculate new amplitude of each electrical spectral line applied to the DAC:


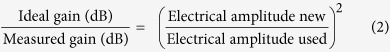


After 3–10 iterations (related to the shape precision) of the digital feedback compensation^45^, we can obtain the long-term stable pump waveform for arbitrary-shaped filters.

In order to mitigate the incalculable gain induced by four wave mixing (FWM) effect among the multiple pump lines, we further set the frequency interval of the electrical spectral lines randomly around the natural SBS gain bandwidth instead of the equal interval. In this case the FWM induced gain will no longer superpose on the original lines and the feedback process is more accurate. Note that once the feedback compensation is completed, the optimal pump waveform is fixed and can be stored for future use.

The proposed filter can be considered as a fully software-defined MPF. Currently the whole arbitrary-shaped pump generation and feedback process can be realized by automatic software control. This lays the root for systematic integration and instrumentation. In this paper we focus more on the bandpass filter as an example. It should be noted that the filter can be either bandpass with SBS amplification or band-stop with SBS absorption.

### Arbitrary-shaped MPF demonstration

The experimental setup is shown in Fig. 1b. An external cavity laser (ECL) operating at 1550 nm is split into two branches for the generation of pump and probe signal in the upper and lower branch respectively. By using a single laser source we can achieve more stable measurement and conduct accurate pump control^51^. In the upper branch, a DAC is controlled by a signal processor and generates designed electrical spectral lines with random frequency interval within ±1-MHz deviation from the natural SBS bandwidth of 20 MHz, (i.e. 19 MHz, 20 MHz and 21 MHz). Then it modulates the CW light to generate the optical carrier-suppressed single-sideband (OCS-SSB) SBS pump lines utilizing an I&Q modulator (IQM). Thus each frequency line of the pump can be accurately controlled. After boosted by a high power erbium-doped fibre amplifier (EDFA), the OCS-SSB signal is sent into a 25-km long single mode fibre (SMF). A polarization controller (PC) is used to maintain the SBS gain at the maximum value. The SBS gain is ~11-GHz downshift from the pump as shown in Fig. 1b(i). In the lower branch, a sweeping signal covering the whole SBS gain region from an electrical vector network analyser (EVNA) modulates the CW light utilizing a Mach-Zehnder modulator (MZM) to generate the probe signal. After suppression of the left sideband by an optical bandpass filter (BPF) as shown in Fig. 1b(ii), the probe light propagates through the fibre and is amplified once it has swept within the SBS gain region as shown in Fig. 1b(iii). Then the probe signal is detected by a photodiode (PD) and sent into the EVNA. The amplitude response sketched in Fig. 1b(iv) is measured by the EVNA and the SBS gain spectrum can be obtained by comparing the results between the SBS pump on and off.

The proposed filter has multi-dimensional flexibility and can be tuned with very high precision. The tuning speed is now limited by the switching time of the DAC output waveform. The theoretical switching time equals the time duration in which the pump propagates through the whole fibre. If the fibre is ~20 km, the switching time is merely ~100 us.

By adjusting the frequency of a single electrical spectral line accurately therefore changing the pump frequency, the filter central frequency can be tuned precisely as shown in Fig. 1c,d. Thanks to the narrow linewidth of Brillouin gain, the minimum filter bandwidth can be as narrow as ~20 MHz with a Gaussian profile which corresponds to a single frequency pump^52^. The tuning step in the figure is set to ~1 MHz but theoretically the tuning resolution can be even higher by simply using longer temporal pump waveform to increase the spectral resolution. This precise central frequency tunability can dynamically compensate the central frequency drift of the RF signal or Brillouin frequency-shift change due to the temperature/stress variation of the fibre^51^.

By altering the number of the electrical spectral lines, the filter bandwidth can be changed. As shown in Fig. 1e,f, we demonstrate the filter bandwidth tunability by changing the number of pump lines from 1 to 10. The filter bandwidth increases with a step of ~20 MHz, when the pump lines are more than 3. More precise tuning can also be realized by slightly changing the position of the frequency lines at both edges. The tuning can range from ~20 MHz to several GHz thus making it capable of dealing with RF signals of different bandwidth^43^. In addition, from Fig. 1f we can see that the in-band phase variation is related to the filter bandwidth. Therefore we can also vary the phase shift of the RF signal by using the MPF with different bandwidths. The minimum bandwidth limitation is from the Brillouin natural linewidth, which can be decreased to ~3 MHz by inducing Brillouin loss on both edges of the Brillouin gain^53^. The maximum bandwidth limitation is due to the increasing pump consumption with the increase of the filter bandwidth. In this paper, we limit our demonstration within 3 GHz. However with multi-stage amplification, the Brillouin pump can be used in a more efficient manner and the maximum bandwidth can be further extended^44^.

**Figure 2:**
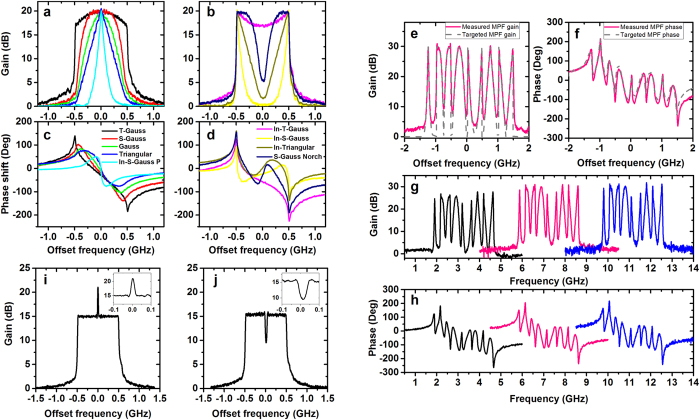
Software-defined arbitrary-shaped MPF. The (**a**,**b**) amplitude and (**c**,**d**) corresponding phase response of MPFs with different shapes defined by software with 1-GHz bandwidth. The measured and targeted (**e**) amplitude and (**f**) phase response of a 3-GHz MPF comprising six 500-MHz sections configured to inverse super-Gaussian peak, Gaussian and triangular shapes followed with their complementary shapes adjacently from left to right. The (**g**) amplitude and (**h**) phase response of the 3-GHz MPF with different central frequency by changing the wavelength of the pump laser. (Note that we use a fixed laser for the probe signal and another tunable laser for the pump in this demonstration.) Fine filter shaping adjustment with (**i**) a single peak or (**j**) notch.

Except for the filter tunability of central frequency and bandwidth, the filter shape can also be defined precisely. This is the largest advantages of the proposed MPF against other approaches. By generating different pump spectra and applying the feedback compensation according to the targeted filter shape, the final MPF shapes are obtained as shown in Fig. 2a–d, including truncated-Gaussian (T-Gauss), triangular, inverse super-Gaussian peak (super-Gaussian shape turned upside down then left and right reversed, marked as “In-S-Gaussian P” in the figure), inverse triangular (In-Triangular) and so on. The corresponding phase responses are also different, which provide approaches to signal phase control. But it should be noted that the filter phase response is always dependent on the filter amplitude response.

We can not only realize symmetric filter shapes but also random combinations of these shapes. In Fig. 2e,f, we demonstrate the amplitude and phase response of a 3-GHz MPF comprising six 500-MHz sections which are configured to inverse super-Gaussian peak, Gaussian and triangular shapes followed with their complementary shapes adjacently from left to right. The measured gain is slightly different from the targeted shape due to the uncontrollable FWM of dense pump lines. In order to tune the filter central frequency in a larger scale, we use two lasers for the pump and probe signal respectively and tune the wavelength of the pump laser with 4-GHz shift step while keeping the probe laser frequency fixed as shown in Fig. 2g,h. The filter amplitude and phase responses are well kept with no need for further feedback. Literally we can realize a filter with arbitrary shape with resolution of ~MHz within ~3 GHz bandwidth.

Finally we show the capability to vary the filter shape at specific single frequency while keeping the rest part fixed in Fig. 2i,j. By only altering the amplitude of the corresponding electrical spectral line, a peak or a notch is realized at the very centre of the rectangular filter, which again proves the high filter resolution. It should be noted that the defined resolution is limited by the natural linewidth of SBS effect. With the increase of the peak gain, the defined resolution will be lower due to the broadening of the overall peak width (calculated at the rectangular pedestal). Similarly, when the notch is deeper, the overall notch width will also be larger.

### Polarization insensitive MPF

It is well known that the SBS gain is polarization dependent, which means that the proposed filter in last section is varying based on the state of polarization (SOP) of the signal. By using specially designed depolarized frequency-sweeping pump, we have realized a polarization insensitive filter^47^. Moreover, the sweeping pump will eliminate the four wave mixing in multi-tone pump scheme thus reducing the control error and contributing to a more precise filter shape. Here we demonstrate that the polarization-independent arbitrary-shaped MPF can also be realized based on depolarized frequency-sweeping pump.

**Figure 3:**
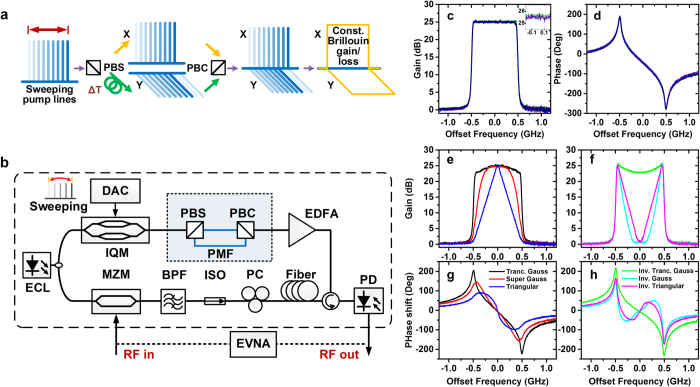
Polarization insensitive arbitrary-shaped MPF. (**a**) Principle of polarization-independent SBS by using depolarized frequency-sweeping pump. (**b**) Experimental setup of polarization-independent arbitrary-shaped MPF generation. The (**c**) amplitude and (**d**) phase response of the polarization insensitive rectangular filter with probe at 5 different SOP. The (**e**,**f**) amplitude and (**g**,**h**) corresponding phase responses of polarization insensitive arbitrary-shaped filters.

The principle of Brillouin pump depolarizing process is shown in Fig. 3a. First we use a linear frequency-sweeping signal as the pump, which is modulated by an electrical sweeping signal from the DAC as used in the multi-tone pump case. As long as the pump propagation time through the fibre is much longer than the duration of the sweeping cycle, the pump can be considered as a broadband pump^33^. Then the pump wave passes through a polarization beam splitter (PBS) and is separated along two orthogonal polarization states. After time delay in one branch to induce fields decorrelation, the two branches are combined using a polarization beam combiner (PBC). Since there is only a single frequency existing at any specific time for each polarization state and the signal frequency on the two polarization projections are different at all times, the sweeping pumps along the two polarization states are independent. Thus, the pump can be considered as a polarization-multiplexed pump. Therefore the Brillouin gain and loss are both polarization-insensitive.

This architecture is effective for the frequency-sweeping pump scheme. For the multi-tone pump, however, the phase difference varies linearly with the frequency for the same delay. So the phase difference between two polarization projections results in an SOP variation of each spectral line, which makes the filter shape out of control. For polarization-insensitive operation in the multi-tone pump case, polarization scrambler with fast tuning speed is required.

The experimental setup for depolarized frequency-sweeping pump based MPF is similar with the multi-tone scheme as shown in Fig. 3b. The only difference is that the OCS-SSB signal is split by a PBS and then combined together by a PBC after experiencing different delay. In the experiment, we use a 10-m polarization maintaining fibre (PMF) for inducing a time delay of ~50 ns. Correspondingly, the sweeping speed is set to 1 MHz/ns. Thus frequency difference between two branches is ~50 MHz, which is larger than twice of the SBS linewidth to ensure the independence between the two orthogonal polarization states. Actually the sweeping speed is very important and should be chosen carefully taking into account the delay time, Brillouin media length and filter bandwidth.

After precise feedback compensation, we obtain a long-term stable polarization-insensitive SBS gain, as shown in Fig. 3c,d for a 1-GHz wide rectangular Brillouin filter. The feedback iteration times are less compared with that in multi-tone pump case since no FWM happens. No matter how we change the SOP of the probe signal, the filter shape is constant while keeping a fixed and smooth phase response, which proves the validity of the pump depolarization process.

By using the polarization-multiplexed frequency-sweeping pump, we can also define the filter shape with very high precision as in the multi-tone pump case. We demonstrate some typical filter shapes such as truncated Gaussian, triangular, inverse Gaussian etc. in Fig. 3e–h. It should be noted that, compared with the arbitrary shapes realized by multi-tone method in Fig. 2, the sweeping pump method eliminates the out-of-band gain completely, making the shape more precise. Here we only demonstrate filters with 1-GHz bandwidth but they can be easily extended to a larger bandwidth. The MPF selectivity can be improved by using multi-stage configuration.

This depolarization setup provides a polarization insensitive filtering solution and simplifies the system complexity via the reduction of polarization controllers especially when we implement multi-stage filtering architecture. Nevertheless, the orthogonal pump will result in pump redundancy. Thus in order to reach the same Brillouin gain, theoretically the pump power increases by a factor of 3/2 compared with the single SOP pump^54^. Experimentally the increase of the pump vary from 1 to 2 dB depending on the Brillouin gain value and probe signal level.

### Pulse shaping demonstration

In order to prove the feasibility and practicability of the arbitrary-shaped filter, we demonstrate temporal pulse shaping with the proposed filters. After passing through different filters, the pulse shape has been changed due to the spectral transformation.

**Figure 4:**
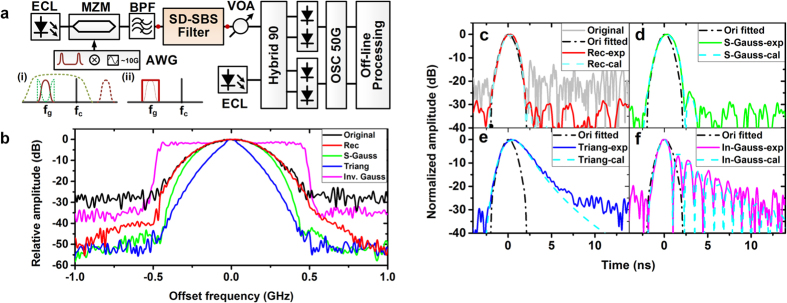
Pulse shaping by using the proposed arbitrary-shaped MPF. (**a**) Experimental setup for pulse shaping. Inset (i) the single sideband spectrum of an upshifted Gaussian pulse before the software-defined SBS (SD-SBS) filter and an inverse Gaussian SBS filter at exactly the same frequency. (ii) the pulse after the SD-SBS filter with a rectangular spectral shape. (**b**) The spectral profiles of the pulse train after passing through different filters. (**c–f**) The pulse shape transformation before and after passing through SD-SBS filters of (**c**) rectangular, (**d**) super Gaussian, (**e**) triangular and (**f**) inverse Gaussian shape. The dashed dot line is the fitted Gaussian pulse shape before filtering. The dashed and solid lines are the calculated and measured pulse shape after filtering.

The setup for pulse shaping is shown in Fig. 4a. An ECL laser is modulated by an electrical Gaussian pulse train which is generated from an arbitrary waveform generator (AWG). In order to avoid the relative laser drift, we use the same laser to generate the pulse train and the pump. Thus the pulse train is upshifted by ~10 GHz inside the AWG. After removed one upper sideband by using a BPF, the pulse train passes through the software-defined SBS (SD-SBS) filter and experiences spectral transformation. Then an optical attenuator is adopted to adjust the signal power level. Finally the pulse train is detected by a typical coherent receiver. Compared with direct detection with pre-amplification, the coherent detection provides better sensitivity.

The width of the Gaussian pulse is ~4 ns with a period of 100 ns, whose spectral profile is ~1-GHz wide. For the pulse shaping, we use four 1-GHz wide filters with the shape of rectangular, super Gaussian, triangular and inverse Gaussian respectively. The frequency responses of the filters have already been shown in Fig. 3e–h. After passing through different filters with 25-dB gain, the spectral profile of the Gaussian pulse train is transformed accordingly as shown in Fig. 4b. The rectangular filter keeps the Gaussian spectrum as it is. The super Gaussian and triangular filters compress the spectrum. The inverse Gaussian filter amplifies only high frequency components and makes the spectrum rectangular. The transformed spectral profiles are all very smooth, which imply that the filter shapes are well controlled. Note that for the inverse Gaussian filter, in order to match the signal power level we adjust the filter gain to ~22 dB.

The spectral transformation is accompanied by the pulse shape variation in time domain. The shape comparison of the Gaussian pulse before and after passing through different filters are shown in Fig. 4c–f. The rectangular filter maintains the original pulse shape very well. The super Gaussian filter broadens the pulse slightly at the falling edge and induces a side lobe which is ~25 dB smaller than the main lobe. The triangular filter obviously broadens the Gaussian pulse at the falling edge and makes it more like a triangular pulse. The inverse Gaussian filter compresses the Gaussian pulse and induces several side lobes after the falling edge, which is consistent with its rectangular spectrum. We also calculate the theoretical filter response by convoluting the natural Brillouin gain with the designed pump spectrum. The experimental results show very good agreement with the calculated results thus proving the high control precision and accuracy again.

Another remarkable advantage by using the proposed filter is the selective amplification. From Fig. 4b in frequency domain and 4c in time domain we can clearly observe that the noise level has been decreased and the signal is smoother due to the 25-dB amplification. The inverse Gaussian filter is an exception since it just amplifies the high frequency components and keeps the central frequency components at the original level.

## Discussion

As we mentioned in the last section, this MPF can also be regarded as an active amplifier. While it provides tens of dB gain for the signal, it will also induce extra noise due to the spontaneous Brillouin scattering^45^,^52^. The higher the filter selectivity is, the larger the pump power will be and thus inducing more noise. In order to optimize the filter performance, the filter parameters should be well chosen such as fibre length, number of stages and so on. Our present work is focusing on improving the noise performance of the filter.

The integration of microwave photonic devices brings advantages in terms of size, weight and power (SWAP) consumption, which has become a trend. Although in this paper we demonstrate our proposed filter with bulk optical components as a proof of concept, it has the potential possibility to be more compact. The essential components of the proposed MPF consist of a laser source, a programmable DAC, an IQM, an EDFA and a section of fibre. The DAC can be integrated in a circuit board with a field programmable gate array (FPGA) and peripheral circuit. For simplicity, the IQM can be replaced by a standard intensity modulator at the cost of slight degradation of control precision. The 25-km fibre can also be replaced by short fibre or waveguide with high Brillouin efficiency^55^,^56^. The feedback calibration is only needed for filter initialization and will not increase the control complexity. Therefore it is possible to make this MPF a desktop instrument. It can also be further integrated considering the current progress of on-chip SBS^34^. Our future work is towards filter compactness and prototype development.

In summary, we have realized a software-defined single passband arbitrary-shaped microwave photonic filters with ~GHz bandwidth based on SBS effect in optical fibre. This method can generate arbitrary-shaped filter with resolution of ~MHz within ~3 GHz bandwidth. Higher bandwidth can be achieved by using multi-stage Brillouin amplification. The filter shape, bandwidth and central frequency reconfigurability have been fully demonstrated with unprecedented precision, which has not been obtained before. By depolarizing the linear frequency-sweeping pump, the filter can be polarization insensitive without any out-of-band gain. Based on this arbitrary-shaped filter, we demonstrate the pulse shaping capability. The filter can broaden or compress the Gaussian pulse and intentionally induce one or more side lobes. The filtered pulse shapes have very good agreement with the targeted shapes proving the high control precision and accuracy. Meanwhile the filter also plays the role of selective amplifier, which can provide tens of dB gain. The multi-dimensional filter flexibility provides precise optical processing approach in microwave photonic fields and makes it a promising MPF solution. Meanwhile the core optical filter technique can also find versatile applications in optical communications and high-resolution optical signal processing.

## Methods

### Brillouin pump generation

We use MATLAB to design the frequency response of the filter and generate the electrical waveform by using a DAC. In this paper we use an AWG (Tektronix 7221, 12-GHz sampling rate and 10 bits resolution) as DAC to demonstrate large bandwidth MPF. We also used an FPGA-controlled DAC board (EUVIS, 10 bits linear resolution, 4 GHz sampling rate) instead of expensive AWG. For generating small bandwidth filters, they performed almost equally. After passing through a 4-GHz anti-aliasing low-pass filter and amplified by an electrical amplifier, the electrical waveform passes through a 90° hybrid coupler to generate two signals with 90° phase difference, which will drive the two child MZMs of the IQM (Photline MXIQ-LN-40) respectively. The three bias voltage values of the IQM are precisely adjusted to realize OCS-SSB modulation and are kept constant day and night for stability. The OSC-SSB optical signal will be boosted to a proper level and be injected into the fibre as the Brillouin pump.

### Feedback process

The filter response is measured by using an EVNA (ROHDE & SCHWARZ ZVK, 10 MHz~40 GHz) covering the whole SBS gain region. First we turn off the Brillouin pump and measure the beating signal between the carrier and the sweeping probe from the PD as the base. Then we turn on the Brillouin pump and measure the signal again as the system response. The amplitude and phase response of the filter can be obtained by subtracting the base from the total system response. The measured response will be processed by MATLAB and the new electrical waveform for the pump will be generated afterwards. In the experiment, all the equipment is connected to a computer via GPIB or Ethernet connection and controlled via MATLAB. Thus the whole feedback process can be done automatically within a short time. For a specific MPF, once the feedback compensation is done, the optimal pump waveform is fixed and can be stored for future use.

## Additional Information

**How to cite this article**: Wei, W. *et al*. Software-defined microwave photonic filter with high reconfigurable resolution. *Sci. Rep.* **6**, 35621; doi: 10.1038/srep35621 (2016).

## Figures and Tables

**Table 1:** Comparison of different kinds of MPFs.

Filter type	Key techniques	3-dB Bandwidth (MHz)	Frequency tuning range (GHz)	Selec-tivity (dB)	Shape control capability	First author and Ref.
BP	Fibre SBS + OCS-SSB + FBC	20–3000	3–11*	57** 47	RCB, RES:~MHz	This paper
BP	Fibre SBS + IM	250–1000	1.65–2.15*	44	RCB	Stern^33^
BP	On-chip SBS + PMS + FBC	30–440	0–30	44	RCB	Choudhary^34^
BP	Fibre SBS + IM	24–45	1–19	23	ND	Vidal^17^
BP	On-Chip SBS + PMS	20–40	2–12	20	ND	Byrnes^25^
BP	Fibre SBS + PMS	21	0–20	30	ND	Hu^23^
BP	Fibre SBS gain & loss + PMS	20	1–20	31	ND	Zhang^22^
FIR	SBS grating	~1000	Within FSR	~10	ND	Sancho^37^
FIR	SBS-assisted multitap	300	FSR/5	12	ND, FSR:2 GHz	Sagues^36^
BS	On-chip SBS + DSC	100–300	0.075–30	20	ND	Aryanfar^30^
BS	On-chip SBS	126	2–8	20	ND	Morrison^57^
BS	Nanowire SBS + DSC	98	14–20	48	ND	Bedoya^28^
BS	On-chip SBS + DSC	33–88	0–30	55	ND	Marpaung^27^
BS	Fibre SBS + DSC	10–65	1–30	60	ND	Marpaung^26^
BP/BS	LC-SLM filtering	500–20000	within 20	~30	RCB, RES: 0.5 GHz	Xiao^16^
FIR	LC-SLM-assisted multitap	~400–700	Within FSR	35	RCB, FSR: 10.4 GHz	Hamidi^11^
FIR	Waveshaper-assisted multitap	1000–3000	2–10	~30	RCB, single passband	Xue^42^
FIR	Mircomirror-assisted multitap	~200-~2200	7.5	~15~40	RCB, FSR:11 GHz	Lee^41^

Note that the listed parameters are demonstrated values. BP: bandpass, BS: bandstop, OCS-SSB: optical carrier-suppressed single-sideband, FBC: feedback compensation, PMS: phase modulated signal, DSC: double sideband cancellation, IM: intensity modulation, LC-SLM: liquid crystal spatial light modulator, ND: not demonstrated, RCB: reconfigurable, RES: resolution.

^*^This is demonstrated value, the only limitation comes from opto-electrical components which is tens of GHz.

^**^The real value is related with filter bandwidth.

## References

[b1] Yao J. Microwave photonics. J. Lightwave Technol. 27, 314–335 (2009).

[b2] Capmany J. & Novak D. Microwave photonics combines two worlds. Nat. Photonics 1, 319–330 (2007).

[b3] Minasian R. A. Ultra-wideband and adaptive photonic signal processing of microwave signals. IEEE J. Quantum Elect. 52, 1–13 (2016).

[b4] Minasian R. A., Chan E. H. W. & Yi X. Microwave photonic signal processing. Opt. Express 21, 22918–22936 (2013). 24104178 10.1364/OE.21.022918

[b5] Capmany J. . Microwave photonic signal processing. J. Lightwave Technol. 31, 571–586 (2013).

[b6] Li L., Yi X., Huang T. X. H., Schroder J. & Minasian R. Spectrum-sliced microwave-photonic filter based on Fourier transform of modified optical spectrum. IEEE Photonic. Tech. L. 27, 1422–1425 (2015).

[b7] Xue X. . Programmable single-bandpass photonic RF filter based on Kerr comb from a microring. J. Lightwave Technol. 32, 3557–3565 (2014).

[b8] Sagues M., Garcia O. R., Loayssa A., Sales S. & Capmany J. Multi-tap complex-coefficient incoherent microwave photonic filters based on optical single-sideband modulation and narrow band optical filtering. Opt. Express 16, 295–303 (2008). 18521161 10.1364/oe.16.000295

[b9] Supradeepa V. R. . Comb-based radiofrequency photonic filters with rapid tunability and high selectivity. Nat. Photonics 6, 186–194 (2012).

[b10] Ortigosa-Blanch A., Mora J., Capmany J., Ortega B. & Pastor D. Tunable radio-frequency photonic filter based on an actively mode-locked fiber laser. Opt. Lett. 31, 709–711 (2006). 16544598 10.1364/ol.31.000709

[b11] Hamidi E., Leaird D. E. & Weiner A. M. Tunable programmable microwave photonic filters based on an optical frequency comb. IEEE T. Microw. Theory 58, 3269–3278 (2010).

[b12] Wang C. & Yao J. A nonuniformly spaced microwave photonic filter using a spatially discrete chirped FBG. IEEE Photonic. Tech. L. 25, 1889–1892 (2013).

[b13] Huang T. X., Yi X. & Minasian R. A. Single passband microwave photonic filter using continuous-time impulse response. Opt. Express 19, 6231–6242 (2011). 21451648 10.1364/OE.19.006231

[b14] Palaci J., Villanueva G. E., Galan J. V., Marti J. & Vidal B. Single bandpass photonic microwave filter based on a notch ring resonator. IEEE Photonic. Tech. L. 22, 1276–1278 (2010).

[b15] Gao L., Chen X. & Yao J. Tunable microwave photonic filter with a narrow and flat-top passband. IEEE Microw. Wirel. Co. 23, 362–364 (2013).

[b16] Xiao S. & Weiner A. M. Coherent photonic processing of microwave signals using spatial light modulators: programmable amplitude filters. J. Lightwave Technol. 24, 2523–2529 (2006).

[b17] Vidal B., Piqueras M. A. & Martí J. Tunable and reconfigurable photonic microwave filter based on stimulated Brillouin scattering. Opt. Lett. 32, 23–25 (2007). 17167571 10.1364/ol.32.000023

[b18] Eggleton B. J., Poulton C. G. & Pant R. Inducing and harnessing stimulated Brillouin scattering in photonic integrated circuits. Adv. Opt. Photonics 5, 536–587 (2013).

[b19] Capmany J., Domenech D. & Munoz P. Graphene integrated microwave photonics. J. Lightwave Technol. 32, 3785–3796 (2014).

[b20] Zhuang L., Roeloffzen C. G. H., Hoekman M., Boller K. & Lowery A. J. Programmable photonic signal processor chip for radiofrequency applications. OPTICA 2, 854–859 (2015).

[b21] Marpaung D. . Integrated microwave photonics. Laser Photonics Rev. 7, 506–538 (2013).

[b22] Zhang W. & Minasian R. A. Widely tunable single-passband microwave photonic filter based on stimulated Brillouin scattering. IEEE Photonic. Tech. L. 23, 1775–1777 (2011).

[b23] Hu S., Li L., Yi X. & Yu C. Ultraflat widely tuned single bandpass filter based on stimulated Brillouin scattering. IEEE Photonic. Tech. L. 26, 1466–1469 (2014).

[b24] Tao R., Feng X., Cao Y., Li Z. & Guan B. Widely tunable single bandpass microwave photonic filter based on phase modulation and stimulated Brillouin scattering. IEEE Photonic. Tech. L. 24, 1097–1099 (2012).

[b25] Byrnes A. . Photonic chip based tunable and reconfigurable narrowband microwave photonic filter using stimulated Brillouin scattering. Opt. Express 20, 18836–18845 (2012). 23038523 10.1364/OE.20.018836

[b26] Marpaung D., Morrison B., Pant R. & Eggleton B. J. Frequency agile microwave photonic notch filter with anomalously high stopband rejection. Opt. Lett. 38, 4300–4303 (2013). 24177078 10.1364/OL.38.004300

[b27] Marpaung D. . Low-power, chip-based stimulated Brillouin scattering microwave photonic filter with ultrahigh selectivity. OPTICA 2, 76–83 (2015).

[b28] Casas-Bedoya A., Morrison B., Pagani M., Marpaung D. & Eggleton B. J. Tunable narrowband microwave photonic filter created by stimulated Brillouin scattering from a silicon nanowire. Opt. Lett. 40, 4154–4157 (2015). 26368735 10.1364/OL.40.004154

[b29] Tanemura T., Takushima Y. & Kikuchi K. Narrowband optical filter, with a variable transmission spectrum, using stimulated Brillouin scattering in optical fiber. Opt. Lett. 27, 1552–1554 (2002). 18026503 10.1364/ol.27.001552

[b30] Aryanfar I., Choudhary A., Shahnia S., Pagani M. & Liu Y. Reconfigurable microwave bandstop filter based on stimulated Brillouin scattering in a photonic chip. *in Conference on Lasers and Electro-Optics, OSA Technical Digest*, paper SF1G.7 (Optical Society of America, 2016).

[b31] Zadok A., Eyal A. & Tur M. Gigahertz-wide optically reconfigurable filters using stimulated Brillouin scattering. J. Lightwave Technol. 25, 2168–2174 (2007).

[b32] Wise A., Tur M. & Zadok A. Sharp tunable optical filters based on the polarization attributes of stimulated Brillouin scattering. Opt. Express 19, 21945–21955 (2011). 22109047 10.1364/OE.19.021945

[b33] Stern Y. . Tunable sharp and highly selective microwave-photonic band-pass filters based on stimulated Brillouin scattering. Photonics Research 2, B18 (2014).

[b34] Choudhary A. . Tailoring of the Brillouin gain for on-chip widely tunable and reconfigurable broadband microwave photonic filters. Opt. Lett. 41, 436–439 (2016). 26907391 10.1364/OL.41.000436

[b35] Sakamoto T., Yamamoto T., Shiraki K. & Kurashima T. Low distortion slow light in flat Brillouin gain spectrum by using optical frequency comb. Opt. Express 16, 8026–8032 (2008). 18545512 10.1364/oe.16.008026

[b36] Sagues M., Loayssa A. & Capmany J. Multitap complex-coefficient incoherent microwave photonic filters based on stimulated Brillouin scattering. IEEE Photonic. Tech. L. 19, 1194–1196 (2007).

[b37] Sancho J. . Tunable and reconfigurable multi-tap microwave photonic filter based on dynamic Brillouin gratings in fibers. Opt. Express 20, 6157–6162 (2012). 22418495 10.1364/OE.20.006157

[b38] Pant R. . On-chip stimulated Brillouin scattering for microwave signal processing and generation. Laser Photonics Rev. 8, 653–666 (2014).

[b39] Merklein M. . Stimulated Brillouin scattering in photonic integrated circuits: novel applications and devices. IEEE J. Sel. Top. Quant. 22, 336–346 (2016).

[b40] Capmany J., Mora J., Pastor D. & Ortega B. High-performance low-cost online-reconfigurable microwave photonic transversal filter. European Conference on Optical Communications 4, 833–834 (2005).

[b41] Lee J. H., Chang Y. M., Han Y. G., Lee S. B. & Chung H. Y. Fully reconfigurable photonic microwave transversal filter based on digital micromirror device and continuous-wave, incoherent supercontinuum source. Appl. Opt. 46, 5158–5167 (2007). 17676127 10.1364/ao.46.005158

[b42] Xue X., Zheng X., Zhang H. & Zhou B. Highly reconfigurable microwave photonic single-bandpass filter with complex continuous-time impulse responses. Opt. Express 20, 26929–26934 (2012). 23187547 10.1364/OE.20.026929

[b43] Wei W., Yi L., Jaouën Y. & Hu W. Bandwidth-tunable narrowband rectangular optical filter based on stimulated Brillouin scattering in optical fiber. Opt. Express 22, 23249–23260 (2014). 25321794 10.1364/OE.22.023249

[b44] Wei W., Yi L., Jaouën Y., Morvan M. & Hu W. Brillouin rectangular optical filter with improved selectivity and noise performance. IEEE Photonic. Tech. L. 27, 1593–1596 (2015).

[b45] Wei W., Yi L., Jaouën Y., Morvan M. & Hu W. Ultra-selective flexible add and drop multiplexer using rectangular optical filters based on stimulated Brillouin scattering. Opt. Express 23, 19010–19021 (2015). 26367564 10.1364/OE.23.019010

[b46] Wei W., Lilin Y., Jaouen Y., Morvan M. & Weisheng H. Polarization-insensitive ultra-selective add and drop multiplexer using rectangular Brillouin-based filters. *European Conference on Optical Communication (ECOC)* paper Th.1.5.1 (2015).

[b47] Yi L. . Polarization-independent rectangular microwave photonic filter based on stimulated Brillouin scattering. J. Lightwave Technol. 34, 669–675 (2016).

[b48] Mamdem Y. S. . Importance of residual stresses in the Brillouin gain spectrum of single mode optical fibers. Opt. Express 20, 1790–1797 (2012). 22274523 10.1364/OE.20.001790

[b49] Agrawal G. P. Nonlinear fiber optics. (Elsevier/Academic Press, 2010).

[b50] Boyd R. W. Nonlinear optics. (Academic Press, 2008).

[b51] Nikles M., Thevenaz L. & Robert P. A. Brillouin gain spectrum characterization in single-mode optical fibers. J. Lightwave Technol. 15, 1842–1851 (1997).

[b52] Boyd R. W., Rzaewski K. & Narum P. Noise initiation of stimulated Brillouin scattering. Phys. Rev. A 42, 5514–5521 (1990). 9904689 10.1103/physreva.42.5514

[b53] Preussler S., Wiatrek A., Jamshidi K. & Schneider T. Brillouin scattering gain bandwidth reduction down to 3.4 MHz. Opt. Express 19, 8565–8570 (2011). 21643107 10.1364/OE.19.008565

[b54] van Deventer M. O. & Boot A. J. Polarization properties of stimulated Brillouin scattering in single-mode fibers. J. Lightwave Technol. 12, 585–590 (1994).

[b55] Tow K. H. . Relative intensity noise and frequency noise of a compact Brillouin laser made of as38se62 suspended-core chalcogenide fiber. Opt. Lett. 37, 1157–1159 (2012). 22466180 10.1364/OL.37.001157

[b56] Pant R. . On-chip stimulated Brillouin scattering. Opt. Express 19, 8285–8290 (2011). 21643078 10.1364/OE.19.008285

[b57] Morrison B. . Tunable microwave photonic notch filter using on-chip stimulated Brillouin scattering. Opt. Commun. 313, 85–89 (2014).

